# Career orientation among medical students in Germany: perceived motivating factors and barriers to pursuing oral and maxillofacial surgery - a nationwide survey

**DOI:** 10.1007/s10006-026-01597-7

**Published:** 2026-07-07

**Authors:** Lisa Joana Fleck, Philipp Becker, Frederic Bouffleur, Nicolas Haverkamp, Lukas Benedikt Seifert, Florian Recker

**Affiliations:** 1 University Hospital of Bonn, Venusberg Campus 1, 53127 Bonn, Germany; 2 Department of Oral and Maxillofacial Surgery, German Armed Forces Central Hospital, Rübenacherstr. 170, 56072 Koblenz, Germany; 3 Department of Oral and Maxillofacial Surgery, University Hospital Heidelberg, Im Neuenheimer Feld 400, 69120 Heidelberg, Germany; 4 Dean’s Office, Medical Faculty, University of Bonn, Venusberg Campus 1, 53127 Bonn, Germany; 5 Department of Oral- and Craniomaxillofacial Surgery, University Hospital Basel, Spitalstrasse 21, Basel, 4031 Switzerland

**Keywords:** Oral and maxillofacial surgery, Career orientation, Specialty choice, Medical education, Surgical training, Dual degree

## Abstract

**Background:**

Oral and maxillofacial surgery (OMFS) faces increasing challenges arising from evolving healthcare demands, rising case complexity, and ongoing workforce recruitment challenges, necessitating the long-term preservation of specialized expertise. Although training pathways are well established, career trajectories among medical students remain highly heterogeneous. This study addresses the structural recruitment challenge in OMFS in Germany. It focuses on the mismatch between presumably strong interest in OMFS among medical students, and key barriers, particularly dual-degree requirements.

**Materials and methods:**

A cross-sectional, nationwide online survey was distributed by the Young Forum of the German Association of Oral and Maxillofacial Surgery (Junges Forum der DGMKG) across German medical schools to assess factors influencing career decisions for or against OMFS. The questionnaire covered demographic characteristics, career planning, knowledge and practical experience, and perceived stigma related to OMFS.

**Results:**

The study included 108 medical students (69% female; mean age 25 years), including 64% clinical students, 25% preclinical students, and 11% students in their final practical year. The primary outcome was self-reported interest in OMFS as a potential specialty choice. Interest was highest for OMFS (25%), followed by pediatrics (16%) and gynecology and obstetrics (12%). Completion of an OMFS internship and higher self-rated OMFS knowledge were independently associated with greater interest in OMFS, with internship completion associated with more than sixfold higher odds of naming OMFS as the preferred specialty (OR 6.65, *p* = 0.013). Female participants showed lower odds of preferring OMFS in the exploratory multivariable model and were also less willing to pursue a second dental degree (*p* < 0.001). While perception of training burden did not differ significantly by gender, it varied across training phases. Prior dental education showed a strong bivariate association with OMFS interest (Cramér’s V = 0.50). Operative activity and reconstructive surgery emerged as the main motivators, whereas training duration and dual-degree requirement represented the dominant perceived barriers across all training phases.

**Conclusion:**

Within this exploratory sample of medical students, OMFS was frequently named as a specialty of interest. However, structural and environmental barriers may contribute to reduced willingness to pursue specialist training. Earlier OMFS exposure and greater specialty-specific knowledge were associated with increased interest, whereas the dual-degree requirement, prolonged training duration, and financial burden were perceived as barriers. These findings highlight the importance of earlier curricular exposure, structured practical experience, and targeted support strategies to improve long-term recruitment in OMFS.

**Supplementary Information:**

The online version contains supplementary material available at 10.1007/s10006-026-01597-7.

## Introduction

Oral and maxillofacial surgery (OMFS) is a highly specialized surgical discipline encompassing the treatment of craniofacial trauma, odontogenic infections, facial skin tumors, oral malignancies, dentofacial deformities, and complex reconstructive conditions. Increasing numbers of craniofacial injuries and oral pathologies, together with growing complexity of surgical care, have intensified the need for a sustainable future OMFS workforce [1–5].

In several European countries, including Germany, access to specialist training in OMFS requires dual qualification in medicine and dentistry in accordance with Directive 2005/36/EG on the recognition of professional qualifications [6]. This dual-degree pathway is associated with prolonged training duration, substantial financial burden, and delayed career progression, which may represent barriers to entering the specialty. These structural demands are particularly relevant in the context of evolving career expectations among younger generations of medical professionals, who have been reported to place increasing importance on work-life compatibility and career flexibility [7–9]. At the same time, demographic changes within medical education may further influence future recruitment into OMFS. In Germany, women accounted for approximately two thirds of medical students in 2024, raising important questions regarding accessibility and attractiveness of historically male-dominated surgical specialties such as OMFS [2].

Previous studies have demonstrated that OMFS remains comparatively underrepresented in undergraduate medical education, with limited awareness of the specialty and its career pathways potentially contributing to reduced consideration of OMFS as a career option among students. This may consequently affect both interest and willingness to pursue the specialty [10, 11]. In a nationwide survey among dental students in Germany, the perceived duration of training and unfavorable working hours were identified as major reasons against pursuing OMFS [12]. Furthermore, declining interest has been reported in traditional OMFS subspecialties such as oncology, traumatology, cleft, and malformation surgery, whereas interest in aesthetic and orthognathic surgery has increased [13].

While career motivations among dental students and OMFS specialists in Germany have previously been investigated, limited data exist regarding perceptions of OMFS among medical students in Germany. Research addressing structural barriers, specialty exposure, and willingness to pursue the required dual-degree pathway remains sparse [12].

This study therefore aimed to evaluate whether the presumably low willingness to specialize in OMFS despite a comparatively high level of interest reported by medical students may be associated with structural and environmental barriers representing obstacles to entry into the specialty.

## Materials and methods

### Questionnaire and distribution

This study employed a cross-sectional, nationwide online survey design to assess factors influencing career decisions among medical students in Germany regarding the specialty of OMFS. The 23-item questionnaire was developed by using the Evasys survey tool (evasys V10.0 (2601) - Copyright © 2024 evasys GmbH, www.evasys.ukbonn.de). It was based on a review of existing literature on career choice in surgical specialties and adapted to the specific context of OMFS training in Germany. Content validity was established through a structured expert review involving three specialists in OMFS, medical education, and survey methodology. Feedback was incorporated prior to final implementation. No formal psychometric validation was performed, which is acknowledged as a limitation of this study. The reporting of this online survey was conducted in accordance with the Checklist for Reporting Results of Internet E-Surveys (CHERRIES) guideline. The survey was implemented as an anonymous, voluntary open online questionnaire with fixed item orders. No IP addresses or personally identifiable technical metadata were stored. Therefore, repeated submissions by the same participant could not be entirely excluded. Since distribution to the student councils occurred via a mailing list, neither the number of individuals who received the invitation nor the view rate could be determined. Only questionnaires with complete responses to the main outcome variable were included in the analytical dataset.

The survey covered demographic information, aspects of career planning, available medical specialties, and factors considered important for career decision-making. In addition, participants were asked about their knowledge of OMFS and their self-assessed familiarity with the specialty, perceived technical focus areas, and the assignment of various surgical procedures to the surgical specialties of OMFS, plastic, hand and reconstructive surgery (PHS), or otorhinolaryngology surgery (ENT). Furthermore, perceptions of stigmas associated with the specialty, professionally attractive aspects and perceived barriers, reasons for considering OMFS as a career path, practical experiences, and the need for further information and support were assessed. Finally, open-ended questions explored personal impressions associated with OMFS and potential measures universities could implement to promote interest in the specialty. The survey consisted of 8 multiple-choice questions, 5 single-choice questions, 6 open-ended questions, 3 rating-scale questions, and 1 segmented open-ended questions.

### Participants

Eligible participants included medical students at any stage of undergraduate training at German medical schools or hospitals. Dental students and board-certified OMFS specialists were excluded from the survey population. A screening of all councils for medicine in Germany was conducted to reach medical students to participate in the survey. A total of 39 councils were identified, of which 35 were state universities and 4 were private universities. On behalf of the Young Forum of the German Association of Oral and Maxillofacial Surgery (Junges Forum der DGMKG), the survey was distributed digitally via e-mail to all student councils for medicine in Germany in June 2025. A reminder was sent via e-mail in December 2025. Furthermore, medical students were directly contacted and invited to participate in the survey. The survey was subsequently disseminated through internal, semester-specific communication networks. The data collection process was finalized in February 2026.

### Data

The raw data were collected using Evasys survey tool (www.evasys.ukbonn.de) and analyzed, with the calculation of means and standard deviations using the Evasys reporting tool (evasys V10.0 (2601) – Copyright © 2024 evasys GmbH). Statistical analyses were performed using IBM SPSS Statistics (IBM Corp., Armonk, NY, USA) and Microsoft Excel (Microsoft Corporation, WA, USA). For each individual item, we presented total numbers (n/N) and percentages of selections from all participants. The data consisted of 108 completed questionnaires. Descriptive statistics were computed for all variables. Group comparisons were conducted using chi-square tests and Fisher’s exact tests. Effect sizes were reported as Cramér’s V and interpreted as small (V = 0.1) and moderate (V = 0.3). Statistical significance was defined as *p* < 0.05. Sex/gender was self-reported using the categories male, female, diverse, and prefer not to say. Differences by sex/gender and study phase were analyzed in Excel. Several questionnaire items permitted multiple responses (e.g., sources of knowledge about OMFS, activities and areas of expertise associated with OMFS, reasons for and obstacles to pursuing OMFS, resources used to learn about OMFS, and proposed measures to promote interest in OMFS). For these items, results are reported descriptively only. Percentages were calculated using the number of respondents to the item as the denominator rather than the total number of selections and consequently may sum to more than 100%. No inferential statistical tests were performed on multiple-response items. Chi-square tests for group comparisons (e.g., by gender and study phase) were restricted to single-choice items. Missing responses were excluded item-wise, and denominators are reported alongside each result. (Table 1). 

**Table 1:** Summary of the questionnaire. Sub-questions were indicated by italicized format

Questions
Age/ Sex/Gender/ Nationality/ Current study phase/ Previous degree
*If different, what previous degrees have you completed?*
At what stage of career planning are you currently?
Which medical specialty are you currently most interested in?
How important are the following aspects to you when choosing your field of study?
How would you assess your knowledge of the specialty of OMFS
How did you acquire your knowledge of OMFS?
*If different, how did you acquire knowledge of OMFS?*
What activities do you associate with OMFS?
Which areas of expertise in the specialty of oral and maxillofacial surgery particularly interest you?
*If different, which areas of expertise in the specialty of OMFS particularly interest you?*
Which of the following interventions do you primarily classify as belonging to OMFS (1), PHS (2), and ENT (3)? (17 medical interventions to which the numbers 1-3 can be assigned)
How confident do you feel when you are assigning the interventions?
To what extent do you agree with the following statements about OMFS?
What are your reasons for choosing OMFS as a career path?
What obstacles would you consider to be a career in OMFS?
Have you already completed a clinical elective or an internship at OMFS?
Which resources would you use to learn more about OMFS?
What personal impressions do you associate with OMFS?
What measures could universities take to promote interest in OMFS?

## Results

### General data

In total, 108 participants took part in the survey of which 104/108 (95%) provided their age. The range was between 18 and 36 years with an average age of 25 years. 75/108 (69%) were female participants and 33/108 (31%) male participants. German nationality was reported by 87/105 (83%) of participants, 9/105 (9%) reported dual nationality, 4/105 (3%) Austrian nationality, 2/105 (2%) Swiss nationality, and 1/105 (1%) each reported Italian, Polish, and Albanian nationality. Out of 108 respondents, 37 (34%) reported having previously completed a degree. Of these, 11/108 (10%) had completed a dental degree, 9/108 (8%) a bachelor’s degree, 2/108 (2%) a master’s degree, and 3/108 (3%) a doctoral degree. 12/108 (11%) reported holding other degrees. When stratified by year of study, the results demonstrated that 27/108 (25%) of respondents were in their preclinical study phase (1.-2. academic year), 69/108 (64%) in their clinical study phase (3.-5. academic year), 12/108 (11%) in their final practical year. At the time of data collection, 25/108 (23%) of the participants had decided on a medical specialty. 50/108 (46%) had narrowed their choice to a limited number of options, while 33/108 (31%) remained undecided or had not yet made an active decision. Of those who had narrowed their choice to a few specialties, 34/50 (68%) were in the clinical phase, 9/50 (18%) in the preclinical phase, and 7/50 (14%) in their final practical year. The results are summarized in Table 2.

**Table 2:** Baseline characteristics of survey participants (*n = 108*)

Characteristic	Category	*n* (%) *
Age, years† (*n* = 104)	Mean (range)	25 (18–36)
Sex (*n* = 108)	Female	75 (69%)
	Male	33 (31%)
Nationality (*n* = 105)	German	87 (83%)
	Dual nationality	9 (9%)
	Austrian	4 (3%)
	Swiss	2 (2%)
	Italian	1 (1%)
	Polish	1 (1%)
	Albanian	1 (1%)
Previous degree (*n* = 108)	Any prior degree	37 (34%)
	Dental degree	11 (10%)
	Bachelor’s degree	9 (8%)
	Master’s degree	2 (2%)
	Doctoral degree	3 (3%)
	Other	12 (11%)
Stage of training (*n* = 108)	Preclinical (year 1–2)	27 (25%)
	Clinical (year 3–5)	69 (64%)
	Practical year	12 (11%)
Decision on specialty (*n* = 108)	Decided	25 (23%)
	Narrowed to a few options	50 (46%)
	Undecided	33 (31%)
Study phase of those	Preclinical	9 (18%)
who narrowed (*n* = 50)	Clinical	34 (68%)
	Practical year	7 (14%)

* Values are n (%) unless otherwise specified. Percentages are calculated using the number of respondents who provided data for each item (denominator shown in parentheses next to the characteristic) and rounded. † Age summarized as mean and observed range (years)

### Determinants of medical specialty choice

The importance of various factors influencing the choice of specialty was assessed using a Likert-scale question ranging from 1 (unimportant) to 5 (very important). The evaluated factor of career prospects was rated as very important by 53/108 (49%), important by 44/108 (41%), and moderate by 10/108 (9%) (median = 4). Working hours and work-life balance were rated as very important by 50/108 (46%), important by 29/108 (27%), and neutral by 21/108 (19%). No participant rated them as unimportant (median = 4). Income expectations were rated as very important by 27/108 (25%), important by 42/108 (39%), and moderate by 31/108 (29%) (median = 4). Teamwork in clinical practice was considered very important by 45/107 (42%), important by 32/107 (30%), moderate by 18/107 (17%), and a median of 4. Patient contact was also rated highly (median = 4), with 49/108 (45%) considering it very important and 27/108 (25%) important. In contrast, operative activities showed a more balanced distribution (median = 3). Accordingly, 28/108 (26%) rated them as very important, 22/108 (20%) as important, 22/108 (20%) as moderate, 18/108 (17%) as less important, and 18/108 (17%) as unimportant. The possibility of pursuing an academic career was rated more variably (median = 3), with 30/108 (28%) responding neutral, 26/108 (24%) less important, and 22/108 (20%) unimportant, while 20/108 (19%) considered it important and 10/108 (9%) very important. The opportunity to perform aesthetic procedures was rated lower overall (median = 2), with 38/108 (35%) considering it unimportant, 26/108 (24%) less important, 19/108 (18%) neutral, 15/108 (14%) important, and 10/108 (9%) very important.

### Interest in OMFS and influencing environmental conditions

Specialty interest was assessed via an open-ended question with 103 responses. Multiple mentions per participant were permitted, and each was counted individually. Responses were grouped into main fields with subspecialties assigned accordingly. OMFS was named by 25/103 (25%) respondents, followed by pediatrics (16/103, 16%) and gynecology and obstetrics (12/103, 12%). Within OMFS, the primary areas of interest were facial traumatology (60/108, 56%), reconstructive surgery (56/108, 52%), and plastic and reconstructive surgery (41/108, 38%). Because multiple selections were allowed, percentages do not sum 100%.

Overall, 33/108 (31%) participants had completed an OMFS internship or clinical placement. Of those, 9/33 (27%) were male participants and 24/33 (73%) were female participant. 26/33 (79%) were in the clinical phase and 11/33 (33%) already held a degree in dentistry. Self-rated knowledge in this group was very good in 9/33 (27%), good in 10/33 (30%), moderate in 7/33 (21%), and little in 7/33 (21%). None of the participants with practical OMFS exposure indicated a complete lack of knowledge. Among the 25 respondents with an expressed interest in OMFS, 17/25 (68%) had completed an OMFS internship. The remaining 75/108 (69%) participants had not completed an OMFS internship (24/75, 32% male participants; 51/75, 68% female participants) and accounted for 8/25 (32%) of those interested in OMFS. Their self-assessed knowledge was low in 34/75 (45%), absent in 13/75 (18%), moderate in 22/75 (30%), good in 5/75 (7%), and very good in 1/75 (1%).

Participants evaluated statements about OMFS on a five-point Likert scale. The prospect of an additional degree in dentistry was rated low overall (median = 2). Therefore, 64/108 (59%) strongly disagreed, 13/108 (12%) disagreed, 9/108 (8%) were neutral, 10/108 (9%) agreed, and 12/108 (11%) strongly agreed. Of the 22/108 (20%) who could imagine a second degree in dentistry, 13/22 (59%) had completed an OMFS internship, 13/22 (59%) were male participants and 9/22 (41%) female participants. Moreover, 11/22 (50%) were in the clinical phase, and 5/22 (23%) already held a dental degree. Self-rated knowledge was predominantly good or very good, and they accounted for 16/25 (64%) of those highly interested in OMFS. The training pathway was perceived as too long and demanding by 96/108 (89%), with stronger agreement in the clinical phase. Compatibility with private life was rated lower (median = 2). Accordingly, 20/106 (19%) strongly disagreed, 43/106 (41%) disagreed, 31/106 (29%) were neutral, 10/106 (9%) agreed. Of the 2/106 (2%) participants that strongly agreed, men rated it more positively than women. The unique integration of medicine and dentistry received high agreement with a median of 4. Therefore, 57/107 (53%) strongly agreed and 40/107 (37%) agreed. The remaining responses were distributed across neutral, disagree, and strongly disagree categories. Academic development opportunities showed a more moderate distribution. The median scale was 3. In this regard, 42/105 (40%) participants were neutral, 35/105 (33%) agreed, and 19/105 (18%) strongly agreed. Career opportunities abroad were rated with a median of 3. Consequently, 48/104 (46%) participants were neutral, 34/104 (33%) agreed, 16/104 (15%) strongly agreed, 5/104 (5%) disagreed, and 1/104 (1%) strongly disagreed. Reputation within the medical profession showed a balanced distribution with a median of 3. Accordingly, 5/105 (5%) participants strongly disagreed, 15/105 (14%) disagreed, 34/105 (32%) were neutral, 32/105 (30%) agreed, and 19/105 (18%) strongly agreed.

To formally compare groups across single-choice items, chi-square tests of independence were computed for OMFS interest (yes/no, derived from the open-ended specialty question) and for the Likert statements regarding environmental conditions, against self-reported gender (male participants vs. female participants) and training phase (pre-clinical / clinical / final practical year). Likert responses collapsed to three levels (disagree 1–2 / neutral 3 / agree 4–5) to maintain expected cell counts. Effect sizes are reported as Cramér’s V. Cells with a minimum expected count below five were flagged. In those cases, the chi-square approximation should be interpreted with caution. For these comparisons, a two-sided Fishers exact test (Fishers-Freeman-Halton extension for tables larger than 2 × 2) was additionally computed, and these p-values are reported in the supplementary Table 4. The results are summarized in Tables 3 and 4.

**Table 3:** Chi-square tests of association for OMFS interest and environmental-condition Likert items by gender and training phase

	Predictor	*N*	χ² (df)	*p*	Cramér’s V	
OMFS interest (yes/no)						
	Sex/gender (M/F)	108	0.85 (1)	0.357		0.09
	Training phase	108	1.13 (3)	0.567		0.10‡
	OMFS internship (yes/no)	108	19.26 (1)	< 0.001		0.42
	Prior dental degree	108	27.51 (1)	< 0.001		0.50‡
Likert items × Sex/gender (M/F)						
	Willingness to pursue second dental degree	108	10.61 (2)	< 0.005		0.31‡
	Training too long and demanding	108	2.33 (2)	0.311		0.15‡
	Compatible with private life	106	5.25 (2)	0.072		0.22‡
	Uniquely integrates medicine and dentistry	107	0.13 (1)	0.722		0.03‡
	Academic development opportunities	105	11.14 (2)	0.004		0.33‡
	Good career opportunities abroad	104	0.11 (2)	0.946		0.03‡
	High reputation among physicians	105	1.16 (2)	0.559		0.11
Likert items × Training phase						
	Willingness to pursue second dental degree	108	3.08 (4)	0.545		0.12‡
	Training too long and demanding	108	18.69 (4)	< 0.001		0.29‡
	Compatible with private life	106	6.76 (4)	0.149		0.18‡
	Uniquely integrates medicine and dentistry	107	2.90 (2)	0.234		0.16‡
	Academic development opportunities	105	0.63 (4)	0.960		0.05‡
	Good career opportunities abroad	104	5.28 (4)	0.260		0.16‡
	High reputation among physicians	105	0.40 (4)	0.982		0.04‡

χ² (df) = Pearson chi-square test statistic with degrees of freedom. p-values < 0.05 indicate statistically significant associations. Cramér’s V quantifies association strength: 0.1 = weak, 0.3 = moderate, 0.5 = strong. ‡ At least one cell had an expected count below five. Fisher’s exact p-values for these comparisons are reported in Table 4﻿

**Table 4:** Two-sided Fisher’s exact test using the Fishers-Freeman-Halton extension for cells with an expected count below five

Comparison	*N*	χ² (df)	*p* (χ²)	*p* (Fisher’s exact)
OMFS interest (yes/no)				
Training phase	108	1.13 (2)	0.567	0.547
Prior dental degree	108	27.51 (1)	< 0.001	< 0.001
Likert items × Sex/gender (M/F)				
Willingness to pursue second dental degree	108	10.61 (2)	0.005	0.007
Training too long and demanding	108	2.33 (2)	0.311	0.421
Compatible with private life	106	5.25 (2)	0.072	0.066
Uniquely integrates medicine and dentistry	107	0.13 (1)	0.722	0.720
Academic development opportunities	105	11.14 (2)	0.004	0.005
Good career opportunities abroad	104	0.11 (2)	0.946	0.942
Likert items × Training phase				
Willingness to pursue second dental degree	108	3.08 (4)	0.545	0.511
Training too long and demanding	108	18.69 (4)	< 0.001	< 0.001
Compatible with private life	106	6.76 (4)	0.149	0.121
Uniquely integrates medicine and dentistry	107	2.90 (2)	0.234	0.211
Academic development opportunities	105	0.63 (4)	0.960	0.963
Good career opportunities abroad	104	5.28 (4)	0.260	0.198
High reputation among physicians	105	0.40 (4)	0.982	0.989

**Fig. 1:**
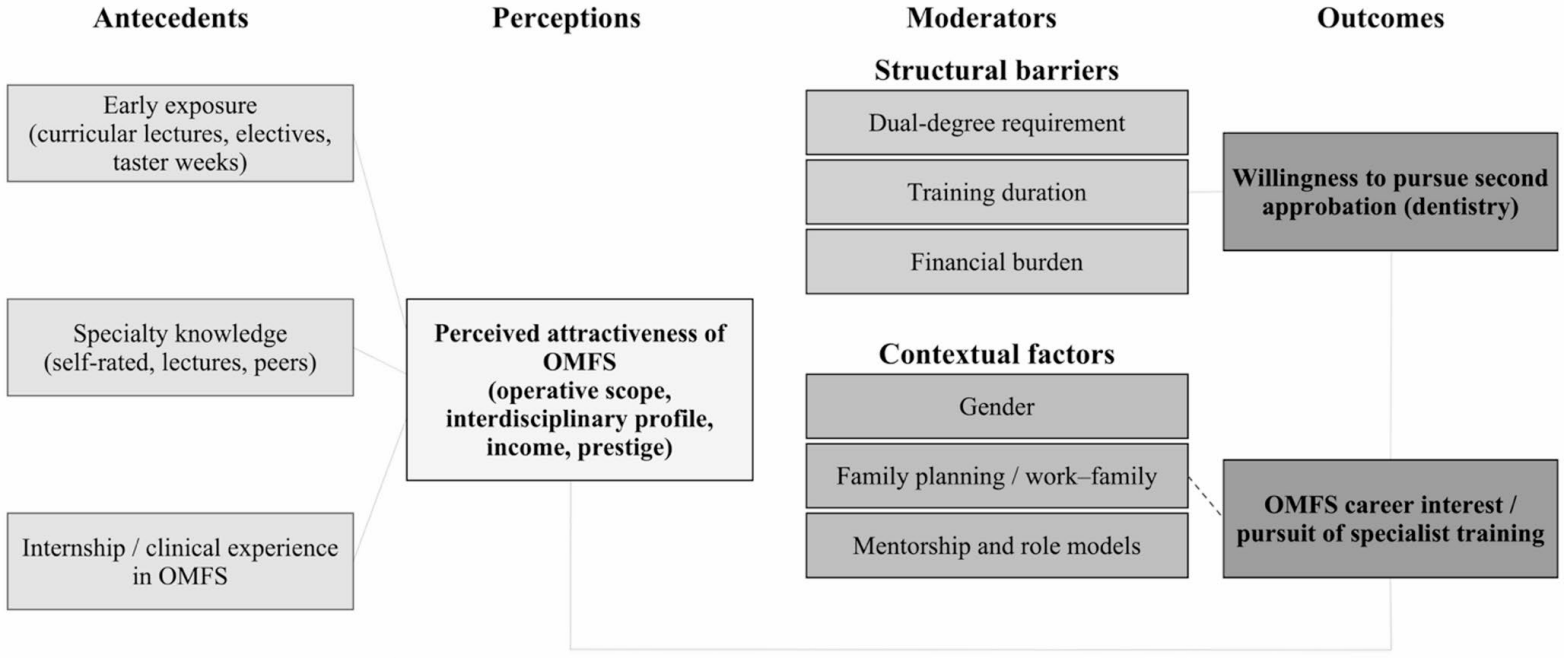
Conceptual model of factors influencing OMFS career interest among medical students

The statistical conclusions of the confirmation by Fisher’s exact test for all comparisons in which an expected cell count was below five, showed no relative changes to the chi-square approximation. As a result, significant associations with interest in OMFS were observed for completion of an OMFS internship and prior dental education, both showing moderate to strong effect sizes. Gender was significantly associated with willingness to pursue a second dental degree, and academic development opportunities. In contrast, no significant gender differences were found regarding the perception of OMFS training as too long and demanding. Perception of training burden, however, differed significantly across training phases. To illustrate the factors influencing interest in an OMFS career among medical students, a conceptual model is presented in Fig. 1.

Solid arrows = direct influence. Dashed arrows = moderating effect. Boxes summarize the main constructs assessed in the present nationwide survey.

To identify independent predictors of OMFS interest, a multivariable binary logistic regression was fitted with OMFS interest (yes/no) as the outcome and gender, training phase (pre-clinical as reference), prior OMFS internship, and self-rated OMFS knowledge (5-point scale, reversed so that higher values denote greater self-rated knowledge) as predictors. The model was based on 108 complete cases (25 with OMFS interest, 83 without) and showed acceptable overall fit (likelihood-ratio test *p* < 0.001; McFadden’s pseudo-R² = 0.36). Given the limited number of outcome events (*n* = 25), the multivariable regression should be interpreted as exploratory. Results are reported in Table 5. Reference categories: male sex, pre-clinical training phase, no prior OMFS internship. Self-rated knowledge was modelled as an ordinal-continuous predictor on the original 5-point response scale.

**Table 5:** Multivariable binary logistic regression for OMFS as the preferred medical specialty (*n = 108* complete cases)

Predictor	Odds ratio	95% CI	*p*
Female (vs. male)	0.33	0.09–1.18	0.089
Clinical phase (vs. pre-clinical)	0.14	0.03–0.70	0.017
Final year (vs. pre-clinical)	0.42	0.06–2.97	0.387
OMFS internship completed	6.65	1.50–29.59	0.013
Self-rated OMFS knowledge (per + 1 step)	2.75	1.53–4.93	0.001

In summary, having completed an OMFS internship and higher self-rated OMFS knowledge were independently associated with substantially greater odds of naming OMFS as the preferred specialty, whereas female sex/gender and more advanced training phase (clinical vs. pre-clinical) were associated with lower odds. However, this finding should be interpreted cautiously due to the limited number of outcome events and wide confidence intervals. The strong bivariate association between OMFS interest and a prior dental degree (V = 0.50) was not entered as a separate predictor because of quasi-complete separation since 10/11 participants with a prior dental degree expressed interest in OMFS.

### Motivations and barriers

When asked about motivators and barriers for choosing OMFS as a career path, respondents could select up to three priorities per question. Values are the percentage of respondents per group selecting the respective item (Table 6).

**Table 6:** Summary of the most frequently selected motivators and barriers for pursuing a career in OMFS, stratified by training phase

Survey item	Pre-clinical (*n* = 27)	Clinical (*n* = 69)	Final year (*n* = 12)
Motivators for choosing OMFS			
Operative activity	52%	55%	58%
Aesthetic / reconstructive surgery	41%	38%	42%
Research opportunities	7%	12%	0%
Interdisciplinary approach	15%	41%	50%
High specialization potential	22%	28%	17%
Good income prospects	41%	42%	33%
Personal interest	44%	36%	50%
Barriers to a career in OMFS			
Duration of training	74%	83%	83%
Dual degree requirement (medicine + dentistry)	74%	67%	58%
Financial burden of additional degree	48%	49%	58%
High workload	11%	39%	42%
Lack of visibility of OMFS during studies	7%	12%	25%
Unfavorable working hours for families	19%	22%	33%
Gender inequalities	11%	13%	0%

**Fig. 2:**
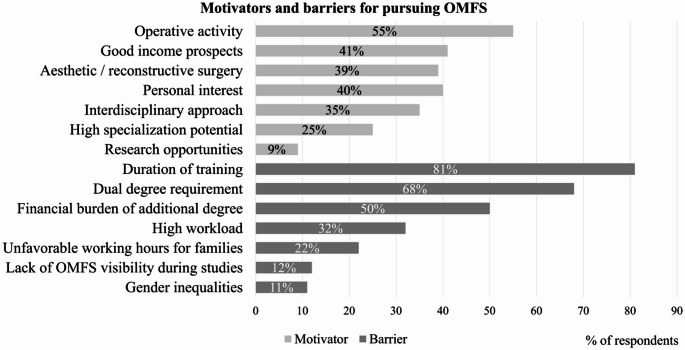
Bar chart of motivators and barriers for pursuing in OMFS as a career path shown as weighted percentages across all training phases (*n = 108*)

Due to the multiple-response format of the items reported in Table 6, the results are presented descriptively only and were not subjected to inferential statistical testing, in accordance with the analysis plan described in the Methods section. Among motivators, operative activity (52–58%), aesthetic/reconstructive surgery (38–42%), good income prospects (33–42%), and high specialization potential (17–28%) were endorsed at comparable levels across all three phases. Larger between-group gradients were observed for interdisciplinary approach (15% pre-clinical vs. 41–50% clinical and final year), and personal interest, which declined from 50% among final year students to 36–44% among pre- and clinical student. Research opportunities were endorsed only marginally throughout (0–12%). Among barriers, training duration (74–83%) and the dual-degree requirement (58–74%) were the dominant items in every group. Larger gradients appeared for high workload (11% pre-clinical vs. 39-–42% clinical and final year), unfavorable working hours for families (19% pre-clinical vs. 33% final year), financial burden of the additional degree (48–58% across student groups), and lack of OMFS visibility during studies (7–12% in early phases vs. 25% in final year). Gender inequalities were a minor concern overall (0–13%). The motivators and barriers across all training phases are illustrated in Fig. 2.

### Information and career promotion

Preferred formats for obtaining more information about OMFS were assessed using a multiple response format, and percentages do not sum to 100%. The most frequently preferred offers were workshops (63/108, 58%), short-term observational placements or trial internships (58/108, 54%), online lectures/webinars, mentoring programs, career counseling (each 28/108, 26%), and research opportunities (18/108, 17%). Regarding delivery formats, participants selected in-person events (72/108, 67%), synchronous online delivery methods (53/108, 49%), written materials (35/108, 32%), and recorded self-directed content (31/108, 29%).

Open-ended impressions of OMFS were provided by 43/108 (40%) participants. They highlighted the specialty as an interesting, diverse, and surgically complex field, while simultaneously emphasizing the prolonged and demanding training pathway associated with the dual-degree requirement. Some participants additionally described the field as hierarchical, male dominated, highly demanding, and characterized by limited teamwork or authoritarian leadership structures. Participants also noted limited curricular exposure to OMFS during medical studies, including minimal teaching integration and low examination relevance.

Suggestions for university-level promotion of OMFS were provided by half of participants (54/108, 50%). Increased curricular visibility through dedicated rotations, seminars, and hands-on practical training was most frequently proposed. Participants further suggested that a shorter, simplified, or better-supported training pathway, including potential modifications to the dual-degree requirement, could improve the attractiveness of the specialty. In addition, respondents indicated that targeted information events, clearer guidance regarding credit recognition, and combined specialty and dental training pathways may facilitate access to OMFS training.

## Discussion

### Principal findings

Ensuring a sustainable pipeline of early-career professionals within each medical discipline is of central importance for maintaining adequate and high-quality healthcare delivery in the long term. As a niche specialty that nonetheless carries substantial medical relevance and clinical complexity, the field of OMFS critically depends on a secure and sustainable recruitment of young professionals.

This study demonstrates that within this exploratory sample of medical students, earlier exposure to OMFS and familiarity with the specialty are associated with increasing interest in pursuing OMFS. Both bivariate and multivariable analyses identified completion of an OMFS internship and higher self-rated OMFS knowledge as major positive predictors of OMFS preference, while prior dental education also showed a strong association with OMFS interest. These findings highlight the importance of early clinical exposure and familiarity with the field. Descriptive analyses further suggested that operative activity, reconstructive surgery, and income prospects represented the most consistent motivators across all training stages, whereas long training duration and the dual-degree requirement were perceived as the dominant barriers throughout all groups. Notably, perceived workload and unfavorable work-life compatibility increased in more advanced training phases, while personal interest in OMFS declined, indicating that increasing clinical experience may alter perceptions of the specialty and its training demands. In contrast, female participants showed lower odds of preferring OMFS and were less willing to pursue a second dental degree, whereas male participants more frequently expressed willingness to undertake this additional qualification. However, this finding should be interpreted cautiously due to the limited number of outcome events and wide confidence intervals. Interestingly, the perception of OMFS training as too long and demanding did not differ significantly between genders, suggesting a comparable evaluation of training burden among male participants and female participants. However, this perception varied across training phases, indicating that increasing clinical experience and progression through training may influence how demanding the OMFS pathway is perceived. Other factors, including international career opportunities and the perceived professional reputation of OMFS among physicians, were viewed similarly across participant groups. To date, there is limited research on medical students’ decisions to pursue additional dental training, and few structured pathways or guidelines exist for integrating such training into postgraduate career trajectories [14].

### Interpretation through social cognitive career theory

The observed mismatch between interest and career intentions may be interpreted within the framework of Social Cognitive Career Theory (SCCT), which posits that career decisions are shaped not only by interest but also by perceived barriers and contextual factors [15]. Although OMFS was consistently reported as attractive because of its operative activity, interdisciplinary scope, and specialization potential, interest alone did not necessarily translate into willingness to pursue the specialty. Instead, structural requirements mentioned by the participants, such as dual degrees, financial burden, perceived work-life compatibility, and reported workplace characteristics, may be associated with how interest is translated into career intentions. Importantly, the cross-sectional design precludes conclusions regarding causality. Nevertheless, these findings appear to support the assumption that contextual and environmental influences substantially shape specialty decision-making in OMFS.

### Role of exposure and specialty familiarity

Interest in OMFS among participants of this study was strongly associated with prior exposure to the specialty, particularly completion of an OMFS internship and greater self-rated OMFS knowledge, suggesting that familiarity and clinical exposure may facilitate career consideration. However, participants reported limited curricular visibility and insufficient structured exposure during medical education, potentially indicating that a lack of early integration may further reduce accessibility of the specialty. These findings are consistent with previous studies in neurosurgery, cardiothoracic surgery and orthopedic surgery suggesting that early exposure programs may shape students’ interest in the fields and therefore enhance recruitment and retention within surgical specialties [16–18]. Accordingly, prior studies from the UK specifically addressing OMFS have similarly reported limited exposure to the specialty within undergraduate medical curricula, insufficient awareness of OMFS conditions and career pathways, and inadequate familiarity with appropriate referral patterns. Collectively, these findings further support the potential value of earlier and more structured integration of OMFS into undergraduate medical education, which may be associated with greater interest in the specialty and willingness to consider OMFS as a career path [10, 11].

### Structural barriers: dual degree, duration, financial burden

Structural aspects of OMFS training emerged as major perceived barriers among participants across all training phases. In particular, the dual-degree requirement, prolonged training duration, and associated financial burden were consistently identified as obstacles potentially limiting pursuit of the specialty. Regarding this, advanced trainees perceived the OMFS pathway as progressively demanding and less compatible with work-life balance, irrespective of gender. Overall, these findings may reflect changing career expectations among younger generations, who increasingly value work-life integration alongside financial and professional attractiveness [19]. Given the identified structural and financial barriers, respondents advocated for financial support, recognition of dual licensure within physician compensation systems, and streamlined training pathways to improve the attractiveness of an OMFS career. However, removing the dual-licensure requirement may limit international recognition in countries where dual qualification remains mandatory [6]. Furthermore, increased outreach to dental students has also been proposed. Nevertheless, this approach would not necessarily shorten training because of the sequential structure of medical and dental education. German regulations further favor a medical-first pathway, as some regions require dual licensure before specialist training, whereas others permit parallel completion of dental studies during OMFS training [5].

### Gender-related implications

Canadian studies underlined the need for gender equality in surgical fields. Gender discordance between surgeon and patient can negatively affect outcomes [20], and female surgeons show slightly lower but significant 30-day mortality with comparable outcomes versus male surgeons [21]. Additionally, medical students in the present cohort described hierarchical structures, limited teamwork, and a traditionally male-dominated working environment as factors potentially discouraging career consideration, especially among women. Moreover, female participants were significantly less willing to pursue the additional dental degree. Comparable findings have also been reported in studies on surgical career choice, where lower pursuit of surgical specialties among women has been linked to work-family conflicts and family-planning-related career interruptions, emphasizing the need for more adaptable training and working structures [15, 22]. A systematic review by Trinh et al. supports these findings, identifying mentorship, intellectual challenge, and early surgical exposure as important motivators for pursuing surgical careers, while gender discrimination, lifestyle concerns, and societal expectations represented major barriers. The authors further emphasized the importance of supportive training structures, parental leave policies, mentorship, and targeted outreach programs for women interested in surgical specialties [23].

### Educational and policy implications

To enhance the visibility of OMFS, participants suggested earlier curricular integration. Supporting this, a nationwide survey of undergraduate OMFS training in Germany found that OMFS is less prominently integrated into medical than dental curricula, particularly regarding formal lectures and structured practical training. The authors therefore advocated for stronger curricular integration and more structured examination formats to improve undergraduate medical education in OMFS [24]. Supporting this, UK medical graduates who later pursued an additional dental degree had often gained prior exposure to OMFS through undergraduate electives, special study modules, or taster weeks before committing further training [14].

Beyond curricular reform, participants called for more targeted information events and clearer access to structured information on OMFS training pathways, alongside mentorship and career counselling and online lectures or webinars by motivated OMFS specialists acting as role models. The British Association of Oral and Maxillofacial Surgeons (BAOMS) maintains a dedicated website that provides concise information and supports trainee recruitment, complemented by mentoring and support programs (MSPs) that can run alongside medical or dental studies [7]. Similarly, the Young Forum of the German Society for Oral and Maxillofacial Surgery (Junges Forum der DGMKG) serves as a representative body for residents and trainees in OMFS as well as for physicians in specialty training. It also functions as an information network facilitating the exchange of information on clinical, scientific, educational, and social aspects of postgraduate medical training. Moreover, it offers a dedicated section on the main website of the German Society for Oral and Maxillofacial Surgery (DGMKG), where medical and dental students and graduates interested in OMFS, or postgraduate training can submit questions and receive guidance [5].

Based on the present findings, recruitment strategies should focus on three levels. First, the earlier curricular visibility of OMFS in undergraduate medical education is important. Second, structured low-threshold exposure through workshops, taster placements, and mentored clerkships. Third, transparent information on dual-degree pathways, financial implications, and possible training models. These measures may help students translate initial interest into realistic and informed career planning.

### Limitations and strengths of the study

This study has several limitations. First, the questionnaire was only expert pre-tested rather than formally validated, which may affect reliability. Second, the relatively small sample size and unknown response rate may limit generalizability and introduce sampling bias, and several subgroup analyses rest on small numbers, limiting their robustness. Third, about two thirds of participants were female. Although this mirrors the current demographic of German medical students, it may underrepresent male participants perspectives, which is particularly relevant in a still male-dominated field such as OMFS. Fourth, voluntary participation raises the risk of self-selection bias. Students with a pre-existing interest in OMFS or surgery may have been more likely to take part. Potentially overestimating overall interest and responses may be subject to social desirability bias. Together with the study’s exploratory nature and limited external validity, this further restricts generalizability. Fifth, due to the cross-sectional design, causal direction cannot be inferred. Students with a pre-existing interest in OMFS may have been more likely to complete an OMFS internship or actively seek specialty-specific information. Sixth, multiple-response items were analyzed descriptively only. Chi-square testing was confined to single-choice items. No correction for multiple testing was applied, and reported p-values should therefore be read as hypothesis-generating rather than confirmatory. Finally, because OMFS training pathways vary internationally, and no standardized global model exists, findings may not transfer fully to countries without a dual-licensure requirement, where career-choice trends and perceived barriers may differ.

Despite these limitations, the study offers valuable exploratory insights into medical students’ perceptions of OMFS while accounting for the needs of newer generations and ongoing gender shifts. A particular strength given the currently sparse literature in this area, especially in Germany. The findings identify key barriers affecting recruitment and provide insights into the factors influencing medical students’ decisions to pursue a career in OMFS.

## Conclusion

Overall, the findings indicate that despite substantial interest in OMFS among the exploratory sample of medical students in Germany, willingness to pursue specialist training remains limited. Prior OMFS exposure and greater specialty-specific knowledge were associated with increased interest, underscoring the importance of early clinical exposure and familiarity with the specialty. In contrast, structural factors including the dual-degree requirement, prolonged training duration, financial burden, workload, and work-life compatibility concerns were frequently perceived as barriers influencing career decisions. Female participants were less willing to pursue an additional dental degree despite similar perceptions of training burden between genders, suggesting that broader structural and environmental factors may contribute to career decision-making. Taken together, these findings highlight the importance of earlier curricular integration of recruitment strategies including structured practical exposure, and transparent information on training pathways and financial implications. Overall, these measures may support more informed and realistic career consideration in OMFS.

## Electronic Supplementary Material

Below is the link to the electronic supplementary material.

Supplementary Material 1 (DOCX 252 KB)

## Data Availability

The datasets generated and analyzed during the current study are not publicly available due to ethical and data protection considerations related to survey data, but are available from the corresponding author upon reasonable request.
