# Supplementary material for: Career orientation among medical students in Germany: perceived motivating factors and barriers to pursuing oral and maxillofacial surgery - a nationwide survey
Source: Oral Maxillofac Surg. 2026 Jul 7;30(1):107. doi: 10.1007/s10006-026-01597-7 (PMC13341896; doi:10.1007/s10006-026-01597-7)
Supplement: Supplementary file 1 — Supplementary Material 1 (DOCX 252 KB) [file 10006_2026_1597_MOESM1_ESM.docx]

## **Supplements**

Questionnaires used in the study (English translations)

Note to readers

The two questionnaires reproduced below were administered in German. The English versions presented here are professional translations prepared for this Supplementary Material. They were not used for data collection. Item wording, response options, and section structure mirror the original instruments. Likert response anchors have been standardized for readability; intermediate scale points (2, 3, 4) were unlabeled in the original and remain unlabeled here.

Throughout, oral and maxillofacial surgery is abbreviated OMFS. In Germany, board certification in OMFS requires dual qualification in human medicine and dentistry. This institutional context is relevant when interpreting items that refer to prior or additional dental training.

### **Supplement 1. Survey of Medical Students**

*Original German title: „Befragung der Medizinstudierenden – Berufliche Entwicklung von Medizinstudierenden“*

Informed consent

**Consent to participate in the survey**

Please read the following information carefully before starting the survey.

**Aim and purpose of the study**

This study examines the career orientation of medical students in Germany, with a particular focus on oral and maxillofacial surgery (OMFS).

**Voluntary participation**

Participation in the survey is voluntary. You may discontinue the survey at any time without giving reasons and without any disadvantage to you.

**Anonymity of data**

The survey is fully anonymous. No personally identifiable or traceable data are collected. Your responses cannot be linked to you personally.

**Duration**

Completing the survey takes approximately 5–10 minutes.

**Contact**

For questions about the study, you may contact the study coordinators at any time: Mrs. Lisa Joana Fleck (e-mail: [s03lflec@uni-bonn.de](mailto:s03lflec@uni-bonn.de)).

**Consent statement**

“I have read and understood the information regarding participation. I consent to anonymous participation in the survey.”

Section 1. Demographic information

**1.1 How old are you?**

*[free-text response, in years]*

**1.2 Sex**

• Male

• Female

• Diverse

• Prefer not to say

**1.3 Nationality**

*[free-text response]*

**1.4 Current stage of training**

• Pre-clinical phase (years 1–2)

• Clinical phase (years 3–5)

• Final practical year (Praktisches Jahr)

• Graduate / physician in residency training

**1.5 Previous degrees**

• Degree in dentistry

• Bachelor’s degree

• Master’s degree

• Doctoral degree (PhD/Dr.)

• Other

**1.5a If you selected “Other”, please specify the previous degrees you have completed.**

*[free-text response]*

Section 2. Career orientation

**2.1 At what stage of career planning are you currently?**

• Specialty already decided

• Narrowed to a few options

• Still undecided

• No active decision yet

**2.2 Which medical specialty are you currently most interested in?**

*[free-text response]*

**2.3 How important are the following aspects to you when choosing your specialty?**

*Response format: 5-point Likert scale (1 = Not important, 5 = Very important).*

• Career prospects

• Income expectations

• Working hours and work–life balance

• Surgical/operative activity

• Scientific or academic career path

• Teamwork in clinical practice

• Patient contact

• Opportunity to perform aesthetic procedures

Section 3. Knowledge of OMFS

**3.1 How would you rate your knowledge of OMFS as a specialty?**

• Very good

• Good

• Moderate

• Limited

• No knowledge

**3.2 Through which channels have you acquired knowledge of OMFS? (multiple choice)**

• Lectures

• Practical courses (Praktika)

• Clinical clerkships (Famulaturen)

• Internet research

• Personal exchange with OMFS surgeons

• Events (career fairs, workshops)

• Other

**3.2a If you selected “Other”, please specify the channels through which you acquired knowledge of OMFS.**

*[free-text response]*

**3.3 Which activities do you spontaneously associate with OMFS?**

*[free-text response]*

**3.4 Which clinical or surgical areas within OMFS particularly interest you?**

• Facial trauma surgery

• Reconstructive surgery (including microvascular tissue transfer) following trauma or tumour resection

• Craniofacial surgery (e.g. cleft lip and palate, craniosynostoses)

• Orthognathic surgery

• Aesthetic facial surgery

• Dentoalveolar surgery

• Head and neck oncological surgery

• Plastic and reconstructive facial surgery

• Microsurgery and free flap reconstruction

• Surgical treatment of obstructive sleep apnea

• Pre-prosthetic surgery and implantology

• Dermatologic surgery (excision and reconstruction of skin tumors)

• Facial nerve reconstruction

• Other

**3.4a If you selected “Other”, please specify the areas within OMFS that particularly interest you.**

*[free-text response]*

**3.5 For each of the following procedures, please indicate which specialty you primarily associate it with: OMFS (1), Plastic Surgery (PS) (2), or Otorhinolaryngology / ENT (3).**

*Please enter a single number (1–3) for each procedure.*

• Treatment of a mandibular fracture

• Treatment of a midfacial fracture

• Closure of a cleft lip and palate

• Cranial vault remodeling for craniosynostosis

• Bimaxillary corrective osteotomy for skeletal malocclusion

• Blepharoplasty (eyelid surgery)

• Facelift

• Septorhinoplasty (nasal correction with septal correction)

• Avascular bone augmentation in the jaw and facial region

• Resection and reconstruction of a facial skin tumor

• Tumor resection and microsurgical reconstruction of the oral cavity

• Free microvascular flap reconstruction in the head and neck region

• Otoplasty (correction of the ears)

• Correction of facial asymmetries following trauma or malformation

• Facial nerve reconstruction (e.g. reanimation of the facial nerve)

• Functional septal reconstruction following traumatic deviation

• Surgical treatment of obstructive sleep apnoea (e.g. maxillomandibular advancement)

**3.6 How confident do you feel in classifying these procedures?**

*Response format: 5-point Likert scale (1 = Very unsure, 5 = Very confident).*

Section 4. Professional attractiveness and barriers in OMFS

**4.1 Please indicate your level of agreement with the following statements about OMFS.**

*Response format: 5-point Likert scale (1 = Strongly disagree, 5 = Strongly agree).*

• OMFS uniquely combines aspects of human medicine and dentistry.

• The training pathway in OMFS is too long and demanding.

• A career in OMFS offers strong opportunities for academic and scientific development.

• The demands of OMFS are compatible with a good private life.

• OMFS offers good career opportunities abroad.

• I could imagine taking up an additional degree in dentistry.

• OMFS enjoys a high reputation within the medical profession.

**4.2 Which would be your reasons for choosing OMFS as a career path? (please indicate up to three priorities)**

• Surgical/operative activity

• Aesthetic and reconstructive procedures

• Opportunities for research

• Interdisciplinary working style

• Strong potential for sub-specialization

• Good earning potential

• Personal interest

**4.3 Which would be barriers for you to a career in OMFS? (please indicate up to three priorities)**

• Length of training

• Dual qualification required (human medicine and dentistry)

• Financial burden of the additional degree

• High workload

• Limited visibility of OMFS during medical school

• Family-unfriendly working hours

• Gender inequalities

Section 5. Practical experience

**5.1 Have you already completed a clerkship (Famulatur) or practical placement in OMFS?**

• Yes

• No

**5.2 If yes, how would you rate your experience overall?**

*Response format: 5-point scale (1 = Very poor, 5 = Very good).*

• Quality of supervision

• Opportunity for hands-on involvement

• Insight into the breadth of the specialty

• Collegial integration into the team

Section 6. Information needs and support

**6.1 Which offerings would you use to learn more about OMFS? (multiple choice)**

• Workshops (e.g. basic surgical techniques)

• Taster placements (Schnupperpraktika)

• Career counselling

• Online lectures and webinars

• Mentoring programmes

• Research experiences (e.g. laboratory placements)

**6.2 Which information formats do you prefer?**

• In-person events

• Online formats (live)

• Recorded content for self-study

• Written information (brochures, leaflets)

Section 7. Open-ended questions

**7.1 What personal impressions do you associate with OMFS?**

*[free-text response]*

**7.2 Which measures could universities take to foster interest in OMFS?**

*[free-text response]*

**Supplement 2. Pie charts illustrating the relative proportions of surgical procedure**


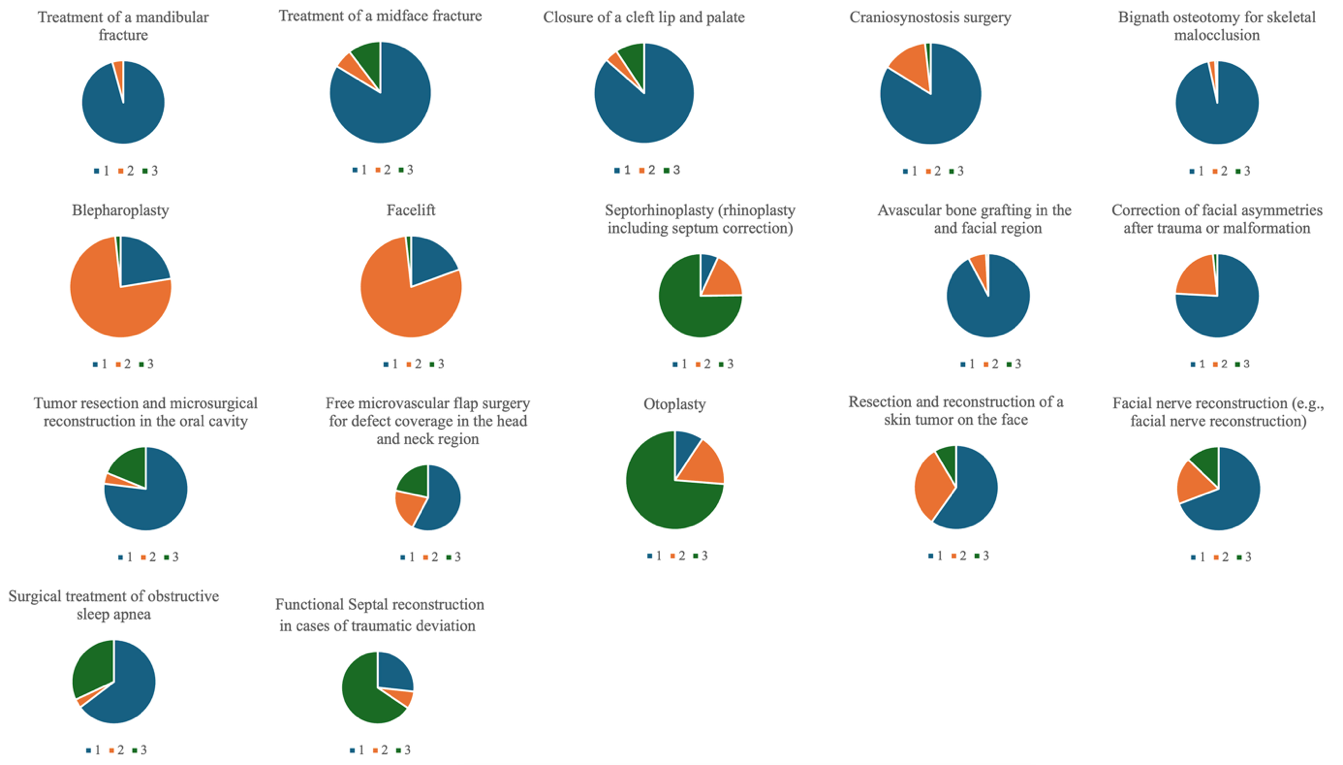


Figure 3 Pie charts illustrating the relative proportions of surgical procedure assignments to oral and maxillofacial surgery (1; blue), plastic, hand and reconstructive surgery (2; orange), and otorhinolaryngology (3; green)
